# Treatise on Hygiène, Public and Individual

**Published:** 1846-01

**Authors:** 


TRAITÉ D'HYGIENE, PUBLIQUE ET PRIVÉE. Par *Michael Lévy*, Professor d'Hygiène Légale a l'Hôpital Militaire de Perfectionnement de Paris. 8vo. Tomes I. & II. à Paris, 1845.

Treatise on Hygiène, Public and Individual. By *Michael Lévy*, Professor of Hygiène. Two Volumes, 8vo. Paris, 1845.

THE present century has been particularly distinguished, both in this country and in many others of Europe, by attempts to determine, through the means of exact and extended observations, the various causes of unhealthiness among large masses of the population. These inquiries have, from the circumstances of the case, principally referred to great manufacturing districts, though the more rural parts have not been altogether neglected. The rapid and unparalleled march of mechanical industry has necessarily attracted vast multitudes together, and one of the results has been, that all the causes of disease, both as relates to individuals and to communities, have received an intense degree of development, and have commanded a correspondent degree of attention. All enlightened Governments have thus felt themselves compelled to inquire into the subject with a view of providing some efficient remedies for evils so widely spread; hence the several Commissions of Inquiry and Acts of Parliament for promoting sanitary improvements which have been appointed and enacted in England, and hence also the Royal Ordonnances and legislative provisions which have appeared during the last few years in France, Prussia, and Austria, for the regulation of labour, and for otherwise ameliorating the health of mechanics and labourers in the great centres of manufactures and commerce.

In order to give a more definite direction to efforts like these, and at the same time to insure a scientific investigation of all the questions which concern the preservation of health, public and individual, professorships have, in several countries, been established, and, among other places, at Paris. That the best results must flow from public lectures on Hygiène, in which would be systematically investigated, in addition to the various modifying circumstances that affect individual life, such as temperament, habit, age, and sex, the more general causes, vitiated atmosphere, bad diet, injurious occupations, unhealthy climates, and so-forth, which operate potentially on the human constitution in a highly civilised state of society, that such an exposition would be beneficial cannot be doubted for a single moment. In England, we believe, there is no special course on this important subject, and yet there is no country in the world where, from the number of manufacturing and crowded districts, it is more urgently necessary to investigate how health may best be preserved, and disease prevented, by sanitary means. In the last number of this Journal an exposition of some of the evil consequences which have sprung from the apathy hitherto prevailing in these matters was given; and we are now anxious to direct the notice of our readers to the treatise recently published by M. Lévy, in which the whole subject of Hygiène is scientifically investigated.

The relations, utility, and objects of this branch of medical science are thus explained by the author:—

"Hygiène is dovetailed, as it were, with all the medical and natural sciences; it is tributary to anatomy, physiology, meteorology, physics, &c.; but it investigates the data which it borrows from them under a particular point of view; thus, whilst physiology considers the organic actions in themselves and in their mutual relations, it is the office of hygiene to examine how these same actions are modified by external agents, and by the reciprocal influence of the organs. The part of chemistry again, is restricted to decompose material substances, and to determine the laws of their combinations; whilst hygiene profits by the inductions which analysis furnishes respecting the effects of these substances, to discover the rules respecting their employment. But if hygiene thus borrows, it also gives; etiology and prophylactics, for instance, repose on it almost exclusively, whilst therapeutics draw from it more resources than from the materia medica. Again, it is impossible to study the effects produced in the constitution of man by the various things which he uses and enjoys, without being conducted to the causes which derange his health; to investigate all that may be hurtful to him is to pass in review all the foci of etiology, whilst to protect him from such injurious agency would be to render useless the intervention of medicine. Even when disease cannot be prevented, the treatment consists more in the judicious application of hygienitic principles than in the administration of special remedies. To regulate the temperature which is proper to the patient, his regimen, his clothing, his morale, is not this, with other similar precautions, the first duty of the practitioner, the essential guarantee for the success of all medication?"—*Tome* I. p. 51.

The author, after favourably noticing the practice of the ancients, who relied as much, or rather more, on the powers of nature than on those of art, proceeds to state, "public hygiene rests on medical statistics and political economy; it constitutes, strictly speaking, the only medicine that is possible for the masses. It only needs a little reflection to perceive that therapeutics generally fail when opposed to epidemic and endemic diseases; that the former, like explosions bursting upon crowded communities, confound the physician, and only allow his art to intervene with advantage in the decline of the affection, when it approaches the character of a sporadic disease. Endemics, again, attacked in detail, only yield to return again with a new energy, and the constitutions which have submitted to these re-iterated attacks, end by becoming deteriorated, in spite of all therapeutic efforts. But where art is powerless to cure, it is often granted to it to preserve; where it cannot hope to stifle the evil, it may succeed at least, thanks to the twofold gift of hygiene, in restricting and attenuating it. Without the rigorous observance of the principles of this science, the vast establishments which Christian philanthropy consecrates to the solace of humanity, would become the seat of desolation and death; and it is only by the same means that the great re-unions of workmen escape the double danger of human condensation and of industrial pursuits. Hygiene is, moreover, the tutelary deity of armies in the field; whilst in peace, watching over the health of the nation, and inspiring the legislature, she gives to the civil government a support which is the more certain, because it is derived rather from the happiness and welfare of the people than from the mere forms and conventionalities of authority."—*Tome* I. p. 52.

In prosecuting the important task which he has undertaken, M. Lévy divides the inquiry into two great branches, according as it affects individuals or numbers, and which he denominates "*Hygiène Privée,*" and "*Hygiène Publique.*"

The first three-hundred pages are occupied with a detailed account of individual peculiarities—temperament, idiosyncrasy, age, hereditary acquirements, habits, &c.; but, as these particulars do not offer much novelty, we proceed to the more important division of the work, which treats of the various external agents capable of affecting the human constitution. Among the most influential of these are what the author, coining a word for the occasion, calls "*modificateurs atmosphériques,*" or atmospheric agents—electricity, light, heat, humidity, and the air itself, as regards its pressure and chemical composition. It is difficult to isolate these, and to attach to each the precise influence it exerts; thus, with respect to solar light, it is combined, independently of the chemical rays, with solar heat, but that it exerts a decided influence on the nutritive actions is shown, among other circumstances, by the paleness of those, who, like the Arabian women of rank, are habitually screened from the rays of the sun. The practitioner accustomed to see the unhappy population of the narrow and dark courts and alleys of great towns will recognize the justice of the following remarks:—

"The individuals who pass a large part of their life in obscure or badly-lighted localities, are not merely distinguished by the character of their skin; their flesh is soft and puffed up, as if infiltrated; they are struck with an atony in all the tissues, and are liable to effusions: such are individuals confined by misery in the most sombre and enclosed quarters of great cities; prisoners confined in gloomy dungeons; sailors whose habitual post is in the hold or store-rooms; porters of houses in Paris situated in the most populous districts; workmen who are employed under ground, &c."

After remarking that these classes are prone to deviations of the osseous system, and corroborating this inference by the assertion of the celebrated Von Humboldt, that "deformity of the body and deviations from the normal standard are extremely rare in certain races of men, especially among the people who have the dermoid system strongly coloured," M. Lévy states that the same observation applies, in a modified degree, in France. "It is (he says) incontestable that the southern population of France presents a conformation more regular and more beautiful than that of the northern, and even in part of the eastern, departments."—*L. c.* p. 341.

With respect to resistance against the depressing influence of cold, this same kind of constitution, characterised by the predominance of the sanguiferous system, by firmness of flesh, by the coloration of the skin, by suppleness of motion, and by gaiety of spirit, is, it is affirmed, more enduring than the opposite or phlegmatic temperament, with its pale and relaxed fibres, its lymphatic aspect, and languid gait. This observation, made by the distinguished Surgeon-in-chief of the Grand Army, Baron Larrey, has been since confirmed by our Arctic voyagers. "I have remarked," says Larrey, "in opposition to the opinion commonly received, that brown persons of a bilioso-sanguine temperament, including almost all the southern countries of Europe, oppose a more energetic resistance to rigorous cold than fair people of a phlegmatic temperament, and generally than northern nations. We have, for instance, seen the Dutch of the third regiment of the Grenadiers of the Guard, composed of 1,787 men, perish almost without exception, for there only returned to France, two years afterwards, 41 soldiers; whilst two other regiments of Grenadiers, consisting almost exclusively of men born in the southern provinces of France, preserved a considerable part of their soldiers. The French, the Portugueze, the Spaniards, and the Italians, are, we again contend, those best adapted to support the vicissitudes of cold and heat in the bivouacs; "a new argument," adds Larrey, "that the inhabitants of southern countries have, in opposition to the assertion of the author of the '*Esprit des Lois*' (Montesquieu), more energy and means of resistance against the action of cold than the people of the North." If all this be true, and it seems probable, it is opposed to all preconceived notions and to the inferences of physiology, for it would certainly appear more probable that Nature would have endowed those branches of the human family with the most active powers of resistance, who, living in high latitudes, are habitually exposed to severe cold.

The author adduces the authority of the late Dr. Edwards, than which none higher could be quoted, to show that the power of generating heat is less in infants than in adults, and even than in old persons. This touches upon a point of moment in the management or hygiene of young children and infants, which is generally too much neglected, both by parents and by medical practitioners. We are satisfied, after extensive observation, that immense mischief results from exposing those of tender age to the rigorous Winters of this country, often insufficiently clad, even among the rich, and not unfrequently with the skin totally unprotected, when older persons are shivering from the cold. It is the duty of our profession to disabuse the public mind of the existing misconceptions on this subject; to make known that all the vital powers of early existence, borrowing from their vivacity a semblance of strength which they do not possess, are intrinsically weak; that, owing to this very vivacity, action and re-action being inseparably proportional, the organic forces are speedily exhausted; that these fundamental laws of animal life can in no degree be abrogated by habit; and that, therefore, the idea of 'hardening' children by an exposure to agencies which the constitution is provided with no means to resist, has no foundation in nature. It would be out of place here to point to the enormous activity of the skin, the large amount of its circulating blood, or to its close and inseparable sympathies with the more purely vital organs of respiration and digestion; nor is it necessary to recal to mind the imminent danger of alarming or fatal congestions of the bronchial and intestinal mucous surfaces, consequent upon checks to the free action of the complex glandular and vascular apparatus lodged in the cutaneous organ.

Although the question relating to the modifications of the atmospheric pressure has but a very limited bearing on hygiene, the following summary of the effects noticed in the ascent of high mountains, by many distinguished savans, will not be devoid of interest.

"These ascents cause a great acceleration of the pulse, a disposition to nausea, a general sentiment of malaise, a lassitude which takes away all force from locomotion, and such an embarrassment of respiration as to render frequent halts indispensable, during which drowsiness comes on. According to MM. Weber and de Humboldt, this muscular lassitude depends in part on the diminished external pressure of the air weakening the support of the thigh in the ileo-femoral articulations. (M. Weber has proved that the atmospheric pressure powerfully supports the articular surfaces in contact, and so diminishes, very materially, the demands on the muscular system.) These phenomena supervene at heights which vary according to the disposition of the individual and the circumstances of the ascent. De Saussure only experienced inconvenience at 11,400 feet, whilst some of his guides, in other respects very robust men, suffered at 9,000, and some even at 5,000 feet. MM. Agassiz and Desor, who ascended the Jungfrau in 1841, did not suffer any inconvenience during a residence of several weeks at a height exceeding 8,000 feet."

A remarkable contrast has been observed in the case of the great American and Asiatic mountains: thus, an English traveller, Moorcroft, found his respiration affected only after ascending a part of the Himalayas 15,600 feet; and Von Humboldt, Boussingault and others, in the ascent of the Andes, did not experience serious inconvenience at a lower range than 17,000 or 18,000 feet. The cause of this striking difference is said to depend on the lower level of the snow-line, which, in Europe, is placed at about 8,000 feet above the sea, whilst in the Cordilleras of the Andes, it is at an altitude of 14,600 feet; but, if the common opinion be correct, according to which the urgent dyspnœa and other symptoms depend on diminished atmospheric pressure, this explanation cannot be well-founded.

The influence of stagnant water in the production of disease is immense, and renders this one of the most important subjects connected with hygiene. The author comprises under this head "all the varieties of water, more or less motionless, which may prove injurious to the health of man by the products of their evaporation—lakes, pools, marshes, salt-marshes, harbours, moats, ditches of fortifications;" and, equally important with any of the preceding, but omitted in the work before us, we may add, all the numberless accumulations in cities, towns, and even villages, which, in the form of puddles, ditches, &c. operate most injuriously. These pestilential places are not confined to the uncultivated and barbarous parts of the world, even where civilization has attained its climax, marshes cover a vast extent of the soil; thus, in France, independently of pools, which abound in many of the departments, it is calculated that they equal 450,000 hectares. (The French hectare equals 2·471143 English acres, that is nearly two and a half acres).

Happily for England the progress of agriculture has, to a great extent, freed it from fens, bogs, and marshes. It is somewhat remarkable that Asia is less infested with marshes than the other parts of the globe, whilst America abounds with them.

The general characteristics of marshy districts are thus graphically sketched:—

"The physical characters of marshes vary according to climate; they also differ in appearance and depth; they have, for a common feature, the development of a certain kind of vegetation, and they further serve as a receptacle for the double products of an organic growth almost boundless in its activity, and of a putrefaction as incessant; mysterious laboratories of life and death, they are at once the cradle and the sepulchre of innumerable generations of plants and animalculæ; they present the contrast of the immobility of their stagnant waters with the agitation of the multitudinous beings which they shelter; and, in order to protect the orgies of an obscene creation, they repulse man, and, by infection and disease, make a solitude around their borders."—*Tome* 1, p. 417.

In general, stagnant waters repose on an argillaceous soil, either naked or covered by a layer, variable in thickness, of vegetable soil, or of mud composed of earthy matters and organic detritus. In some particular instances, impermeability of the soil, arising from other circumstances, is the determining cause, as in the case of the Pontine Marshes, which, according to M. Lévy, arose from the impermeable volcanic tuff forming the soil of Rome and the surrounding country.

With respect to the air resting on marshes (*malaria*), nothing is very certainly known of its chemical qualities, nor even of its exact source. Dr. Dunglison, in his Elements of Hygiene, says, the cause of marsh poison is not known, but that it does not proceed from decomposition of vegetable matter alone, nor of animal matter alone. The late Dr. J. Johnson, whose great practical acquaintance with the subject entitles his opinion to attention, concludes that it is the product of animal and vegetable decomposition resulting from heat and moisture, a conclusion which seems to be the nearest approach to the truth. Many experimentalists have, it is affirmed, even obtained a highly putrescent organic matter from marsh air: thus, Thenard and Dupuytren, by passing it through water, found a deposit of matter, most putrescent in character; Moscati condensed the vapours of a rice-ground, and in a few days the liquid, thus obtained, formed on its surface a mucous substance with a cadaverous odour, analogous to that furnished by the condensation of the exhalations spread in the ward of the great Hôtel Dieu of Milan; lastly, M. Rigaud de l'Isle condensed, by a very simple contrivance, the vapour exhaled by the Pontine Marshes, and he obtained in this way two bottles of liquid, which, being analyzed by Vauquelin, afforded an animal matter. In some of these instances, the gaseous matter was procured from the soil and water of marshes; but Boussingault has obtained organic matter from the air of American marshes, and likewise a large quantity of hydrogen gas. M. Lévy, from whom we have quoted the larger part of the above details, concludes that, "besides the gases which are disengaged abundantly by agitating the water of marshes (light carburetted hydrogen mixed with azote, carbonic acid, sulphuretted hydrogen, and sometimes a trace of phosphuretted hydrogen), it is certain that an organic matter escapes by the volatilisation of stagnant waters, and mixes with their atmosphere ; animal or vegetable, it is without doubt this emanation which determines the specific odour of marshes," and, may we not add, their fatal effects as regards human health.

According to the statistical researches of a high authority, M. Villermé, young children succumb more readily under the influence of marshes than adults. " In comparing the mortality of children in the healthy provinces, and in the eight most marshy departments of France, the proportion is as 1,000 to 1,546. Infants below one year furnish fewer deaths than children of the ages between one and four years, doubtless, because, being kept within doors, they are less exposed to the noxious emanations. After the age of ten years, the influence of marshes is less to be feared; the danger is still less from fifteen and eighteen years up to the age of twenty-five ; from thirty-five or forty years to fifty or fifty-five, the deleterious influence is more pronounced, but never so much as among young children. Old persons, either because they are more sedentary, or because they have acquired the benefit of habit, offer the greatest resistance to the marsh effluvium."

The author, referring to the French settlements in Africa, observes, " it is especially in the time of war that the malaria is fatal to the troops who perform night marches, and who are supplied with food insufficient in quantity, bad in quality, or irregularly distributed. Our military surgeons have acquired an experience of this fact in the campaigns of the Empire, and recently in some localities of Algeria (Bona, &c.), where disease decimates our soldiers. Thanks to the sanitary precautions taken in Africa, and to a good organization of the administrative service, the mortality has diminished there, as it will diminish in all marshy countries by similar ameliorations, of which the most essential consists in a substantial and tonic diet."—(*L. c.* p. 449.) Although as a preventative, generous food is doubtless most beneficial, we entirely dissent from the position just quoted; for it is evident that the most urgent ameliorations required are those connected with drainage, &c., by which means the focus of the mischief is eradicated ; all other precautions are but secondary in their operation.

The article on the Nature of the Soil contains many interesting details, but we have only space for a few extracts. The principal circumstances which bear upon this subject are—1. The mode and degree of exposure, whether to the North or South, &c. ; 2. The meteorological phenomena ; 3. The geological structure, comprising the constitution of the strata, the form of the surface, whether undulated or plain, and so forth ; 4. The relations between the surface of the soil and that of the waters, as rivers, canals, pools, ports, seas, &c.; 4. The existence of forests. M. Lévy remarks that, " the relation of the surface of the earth to that of the waters is an important element of topography ; thus, it is the disproportion of the evaporating surface of water which impresses on so many localities a character of permanent humidity. Venice with its lagunes ; Holland, furrowed by the Rhine, the Scheldt, the Meuse, the Yssel, the Waal, &c. ; Strasburgh, traversed by canals, surrounded by ditches (of fortification), by swamps, by water-meadows, are so many examples of this influence. The mean annual quantity of rain which falls in any locality being known, it becomes important to determine the mode in which it is carried off according to the structure and the configuration of the soil; the amount of fall, the divisions of the streams; the mode of embankment; the system of irrigation established by Nature or by art." The influence of forests on the nature of the soil is, it is well known, to retain the moisture by retarding evaporation; thus, it is stated that Lower Egypt, enriched by the plantation of 20,000,000 of trees, which it owes to Mehemet Ali, receives more pluvial water than Upper Egypt deprived of woods. A contrary effect has followed, in many districts, the destruction of the forests which Spain formerly possessed, owing to the care of the Moors.

The author has, in the following remarks, embodied the results of the best observations on that difficult subject, *Climate*. After noticing the system of the ancient geographers, according to which the space from the Equator to the Pole was divided into thirty climates, and that of the moderns, who partition the same surface into 90°, M. Lévy observes, "the modern division, in multiplying the parallels, has in the same degree increased, for those observers who have applied this arrangement to hygiène, the number of climates. But the influences which in their ensemble characterise a climate, are not distributed in the different regions of the globe, with such rigid regularity, and cannot be reduced to a mathematical classification; neither the meteorological phenomena, nor the conditions of the soil, are identical in all the countries placed on the same parallel of latitude. If, indeed, latitude only were consulted, regions entirely differing from each other as regards their physical characters and the influence they exert on organized beings, would be embraced in the same system of climate; but this word, climate, implies the idea of uniformity, or at least of similarity in the external conditions, so that it is impossible to determine the various climates by purely geographical lines."—(*L. c.* p. 483.) The celebrated Humboldt was the first to substitute for the inflexible parallels of the geographer, a series of isothermal lines, which, resting on the circumstance of an equal mean annual quantity of heat being evolved, and circumscribing the several countries which agree in this respect, describe curves more or less deeply inflected, only preserving their parallelism in the torrid zone. It is not necessary to dwell on the various modifying agencies, which cause the isothermal lines thus to deviate from the parallels of latitude; we need only point out that the most influential of these circumstances are the relative distribution of sea and land, the presence or absence of high mountain ranges, and of extensive forests. Some of the more striking results of these controlling causes are, that Europe enjoys a mean temperature more marked than that of central Asia and of America; that the northern hemisphere receives more heat than the southern; and that, in the same hemisphere, the annual heat diminishes rapidly from West to East in the interior, whilst towards the coast the reverse applies.

The distribution of heat exhibits more striking differences according to the seasons: thus, the latitude being equal, America has more ardent Summers, more rigorous Winters, and more variable intermediate seasons than Asia and Europe; again, in consequence of the more equal temperature of the waters of the ocean, the climate of islands and coasts differs essentially from that of the interior of continents, the former being characterized by mild winters and temperate summers. Besides all these more extensive deviations, there are, in all countries, a vast number of minor causes affecting *localities*, a correct knowledge of which, as Sir James Clark has so clearly shown, is often of the first consequence in considering the influence of climate on health.

In fine, agreeing with M. Lévy that, of all the causes which have a tendency to modify climates, the variations of temperature are the most powerful, we may, with this writer, quote the words of Humboldt, who by "climate" understands "all the modifications of the atmosphere by which the senses are distinctly affected, such as temperature, humidity, the variations of the atmospheric pressure, the tranquillity or commotion of the air, the state of electric tension, the purity of the atmosphere or its mixture with noxious emanations; and, lastly, the degree of habitual transparency, that serenity of the sky so important by the influence it exercises, not only upon the radiation of the earth, upon the development of the organic tissues in vegetables and the ripening of fruits, but also upon the ensemble of the impressions, which, in different zones, are excited by the senses in the soul."

Applied in this comprehensive sense, which is the only true one, it is apparent that, under the term 'climate,' many of the most potential external agents affecting the health and lives of human beings, are comprised. M. Lévy enters into a consideration of the whole subject, but our limits will allow us only to notice one or two of the more interesting points. Among these is that question so often raised :—"is phthisis pulmonalis more rare in hot than in other climates?" After quoting the valuable statistics of the English army, published under the authority of Government by Major Tulloch, and other documents, the author observes, "these numerical data are far from confirming the opinion which attributes to hot climates a favourable influence in respect to the tubercular diathesis; they shew that phthisis exerts its devastating influence with an intensity almost equal in the most different parts of the globe." The difficulty of obtaining a definite solution of the question is shown, and the necessity of taking into consideration many other circumstances besides climate, especially the mode of life, is particularly insisted on. The following extract embraces some details of interest, although it would appear that the ravages of consumption are in some instances underrated. It may be premised that, although there are no sufficient data for estimating the number of persons labouring under phthisis and other complaints in this country, yet, an approximative result may be obtained by ascertaining the proportional number of deaths: from the report of the Registrar-General it appears, for example, that, in the year 1841, there died in the whole of England, 343,847 persons, and of these, 59,592 were cut off by consumption, or somewhat less than one in six; whilst, in London, of 45,507 deaths, 7,326, or rather more than one in six, died from phthisis.

"According to M. Journée, in Leghorn, there is one phthisical patient to forty-four attacked by other diseases; in Florence, one to 28; in Rome, one to 20; in Naples, one to 6. This last calculation probably exceeds the truth, for, according to M. de Rienzi, notwithstanding the influx of invalids, which must, in that city, raise the number of those suffering from tubercle, the proportion of consumptive to other patients is as 1 to 12; an estimate which indicates the superiority of Naples as a place of residence over Paris, where, according to this writer, one phthisical person is met with in four cases of sickness. Lastly, M. Cas. Broussais, who has collected these and other documents in a memoir read before the Academy of Medicine, has made an abstract of the numerical results obtained in the several branches of the medical department in Africa. The medical statistics of fourteen principal stations in the provinces of Algiers, Bona, Oran and Constantine, give one death from phthisis in one hundred deaths, and one case of phthisis in 561 cases of sickness; whilst, according to M. Benoiston, whose researches extend over a period of 12 years, and which are confirmed by those made by M. C. Broussais at the military hospital of Val-de-Grâce, it is proved that, in the French army, one death in five arises from phthisis."

In remarking on these calculations, the author points out two objections which have not been sufficiently considered: firstly, that the soldiers sent to Africa are picked men, the infirm and unhealthy being left in the depôts in France; secondly, that dysentery and destructive fevers, carry off prematurely many individuals, who, at a later period, might have succumbed under pulmonary consumption, so that, one class of disease being substituted for another, the question as regards the protection against phthisis remains undecided.—(*T.* 1, p. 498.)

Intimately connected with the subject above noticed, is the all-important inquiry, how far benefit may be anticipated in phthisical cases from a milder climate. Upon this point M. Lévy observes:—

"Physicians who have practised in hot climates have, for the most part, confirmed the advantages to be derived from them in the case of persons either affected with incipient phthisis, or simply predisposed to this complaint. This important fact has been affirmed by Johnson and Annesley, in the tropical possessions of England; by Twining in Bengal; Segond in Cayenne; Levacher in the French West Indies; Thévenot in Senegal; Gourlay at Madeira; Raymond Faure in Greece; by many of our military physicians in Algiers, &c. Unanimity of opinion like this ought not to be neglected, although it wants the sanction of numerical researches; but we must take care that an advantage, which depends essentially on topographical conditions, is not attributed to entire regions, merely because they belong to the category of hot climates. It is owing to the decisive influence of localities having been mistaken, that so many phthisical patients have submitted to the fatigue of useless migrations, and have gone to seek death where they hoped to find recovery, or at least a prolongation of their days. If the journey into Italy rarely succeeds where the lungs are compromised, it is because Italy is an aggregation of localities which differ singularly by their atmospheric phenomena: every thing depends on making a judicious choice of residence. A short distance, according to the observation of Hippocrates, often changes the whole merit of localities: Nice does not justify the prestige which makes it so much in vogue; Florence ought to be avoided by the consumptive; whilst, at some leagues from thence, Pisa offers a mild temperature without sudden vicissitudes; Genoa and Naples are injurious; the South of France affords but few sheltered retreats, and its Mediterranean coast is to be feared from the frequency of the 'mistral.'"

These remarks, although judicious, cannot claim much originality, for they have been entirely anticipated in the valuable work of Sir James Clark, on the Influence of Climate; indeed, on turning to the leaves of that treatise, it appears to us that our author must have derived all the materials contained in the last part of the above extract from the section headed "Italy" and "Nice." However this may be, the fact of important and influential modifications arising from local causes, must never be overlooked by the practitioner who is consulted in these cases respecting a choice of residence.

A question of much physiological and practical interest, especially in a highly civilized manufacturing country like England, relates to the influence of artificial light on the organ of vision. The great difference between the natural solar light and that of lamps and candles, is, that the one is uniformly diffused and constant, whilst the other is more or less concentrated and varying. "What," asks M. Lévy, "is the action of artificial light upon the visual apparatus? It irritates and fatigues it more than solar light. The watchings and working at night upon very small objects, powerfully contribute to the production of inflammatory diseases of the internal membranes of the eye, to weakness of sight (amblyopia) and to paralysis of the optic nerve (amaurosis). * * * * * * * The intensity of the effects produced by artificial light depends especially on the direct projection of its rays upon the eye, whilst the occupations of the day are carried on in a diffused light. The experience of oculists distinctly proves that an uniform and tranquil light is best adapted to the eye, and that evil consequences result from the agitation of flames; at every oscillation, the eye is forced to alter its focus, and thus fatigue is induced; and in addition the retina, differently affected at each instant, has its powers exhausted." "If the light from a lamp exceeds a certain intensity, the latter should be further removed from the eye; and, as to gas-light, it is too powerful for work. But if too brilliant a light is injurious to the eye, the insufficiency of illumination fatigues the organ by the tension it excites, and by repetition leads to amblyopia and amaurosis."—(2. p. 372-374.) It is stated that the dress-makers suffer severely in Paris, in consequence of being compelled to work in a weak light; in London the same class are similarly affected by protracted labour in brilliant gas-light. In no case, however, are amaurotic diseases more frequently excited than among the mechanics who make stockings, and who are compelled from the lowness of wages to work at night, when they use a strong light produced by a large globe filled with water, which, by collecting and condensing the light, acts as a lens.

We shall conclude our notice of the work before us, by alluding to a subject which is of primary consequence to human health, and which, in an essential manner, is deserving of the deepest attention of our professional brethren: we refer to the construction and ventilation of public edifices. The author remarks, as regards the supply of air (aëration), that "it is proper to distinguish those edifices, such as the cells of prisons, or the wards of a hospital, where the séjour is permanent, or at all events exceeds the duration of a day, from those where the séjour is very limited and does not exceed a night, such as barracks, dormitories of colleges, &c. Buildings constantly inhabited imperatively require the aid of ventilation; it is in fact almost impossible to give them such a capacity as would allow of ventilating means being dispensed with, for, as M. Leblanc judiciously observes, capacity will only delay the moment when ventilation will become necessary."—*L. c.* p. 566.

In the construction of hospitals, a multitude of precautions and rules should be observed, which are, however, frequently violated, but never, as experience teaches, with impunity. In replying to a plan for a hospital submitted to its judgment, the Academy of Sciences " condemned both the circular and the square form, on account of the proximity of the windows towards the interior, allowing the air of one ward to enter into another, and it pronounced in favour of a building in the form of a simple parallelogram, directed from East to West." " As to the number of stories, Hunter, Coste, Pastoret and Villermé have ascertained that, in most hospitals with several stories, the mortality is, cæteris paribus, greater in the upper wards than in the others. The number of patients has a powerful influence on the general salubrity; and it is indisputable that the mortality is much greater in the large than in the small hospitals." It must be regarded as a fortunate circumstance, that in England the number of patients is, comparatively with the great hospitals on the Continent, limited, scarcely ever, in the largest of those institutions in London, exceeding five hundred, and more usually ranging from one to two hundred, whilst in Paris these numbers may be found congregated in a single ward; in La Charité for instance, the wards on the ground-floor and on the first story, contain each from 219 to 225 beds; and, if our memory serves us, some of the wards of the Hôtel Dieu exceed those numbers. M. Lévy truly observes, that " vast wards, well provided with windows, long, lofty or high in the ceiling, please the eye, and are, it is true, much better than narrow and low wards; but the great number of patients which they contain, renders them always more dangerous than small wards which offer the same conditions of aëration and light. Whatever may be the amount of air assigned to each patient, injurious emanations accumulate, and the risk of contagion and infection is in the direct ratio of the population of the beds." Our own experience, resting on an extended examination of the hospitals of this country and on the Continent, entirely confirms the truth of these statements. Small wards, containing, as M. Trousseau advises, not more than twelve patients and even a smaller number, as four or six, combine so many advantages, that the happiest results would flow from their universal adoption: wards of these moderate dimensions allow of the ready renewal of the air, a point, the importance of which it is impossible to exaggerate, but which it may safely be affirmed, has never yet been accomplished in large and crowded wards; they admit of the complete classification of cases; and they tend to the bodily and mental comfort of the patients, by limiting the spectators of those fearful scenes of suffering and death which are so frequently recurring in all large hospitals. M. Lévy's beau ideal, as to construction, and which is said to be realized at the agricultural colony and penitentiary of Mettray, near Tours, is thus sketched: " every hospital should consist of a series of detached buildings (*pavillons*) provided with a ground-floor raised on arches and of a first story, divided into two wards each of 40 beds, and separated by a common vestibule; each building having its offices, a warming apparatus, and a garden or enclosure; lastly, between two of these ' pavillons,' a glass gallery should be provided for the service of the hospital, and for a Winter promenade."—(p. 581). He further recommends that the floor should consist of tiles, or bricks, rather than of flags or boards, which are liable to become impregnated with liquids; that the windows facing North and South, and reaching to the ceiling, should be placed opposite each other, and occupy at least one-third of the whole length of the ward.

These details relating to construction are by no means superfluous, for this is a subject to which the attention of medical men, the only competent judges in such a matter, has been but little directed, at all events in this country. Many of the obvious precautions which are noticed in the foregoing extracts, have been to our personal knowledge neglected both in the metropolitan and provincial hospitals; and that too in cases where the defects were neither dependent on want of funds nor want of space, though of course both these causes, have often, in other instances, led to defective construction. In some of the largest of the London hospitals, the wards are placed opposite to each other, forming quadrangles, the worst possible form, next to the circular, for the free admission of air; in others among them, there are squares within squares, by which the evil of a limited circulation of air, is exasperated; in many, there are windows only on one side of the wards, a most serious defect in the existing absence of efficient ventilation; whilst in others, even the palpable provision of making the windows reach up to the ceiling, is neglected.

The main and essential circumstances connected with the hygiene of hospitals, workhouses, and prisons are, however, those involving the mode of ventilation. Happily the general progress of intelligence, responding to the appeals of philanthropy and benevolence, has wrought great and beneficial ameliorations in the internal economy of these establishments; but it must be confessed that these improvements have resulted rather from the promotion of cleanliness, from the prevention of overcrowding, and from the adoption of measures securing the general comforts of the inmates, than from the application of scientific principles in the removal of the noxious atmosphere invariably generated where numbers of human beings are congregated together. At the time when we write, there are few, if any of those institutions which are more especially committed to the care of our profession, namely, hospitals, in which it can be affirmed that, an effective system of ventilation has been provided. In most cases, this indispensable sanitary condition is trusted, as in private houses, to the insufficient and uncertain method of opening windows and making large open fires; contrivances which, although they may suffice in the case of a single family, can never be applied, except as adjuncts, with any hope of success in the crowded apartments of the sick and diseased. Some attempts have indeed been made in London and elsewhere to secure a more certain supply of fresh air, but being based on unscientific principles, they have not, in general, been attended with satisfactory results. The plan more usually adopted has been as follows: fresh, cold air is introduced by ducts or passages opening into the wards; the impure air is carried off by pipes or flues commencing at the ceiling of the ward and leading out through the roof; and, in some cases, the fresh air, previously to being distributed, is warmed. The general defect is that no certain means are provided for drawing off the foul air *at all times and under all circumstances;* for it should be well understood that the mere insertion of passages leading *directly* into the external atmosphere, offers no security that the stratum of impure air near the ceiling will escape, the question of removal being essentially dependent, under such circumstances, on the relative density of the atmosphere within and of that without. The *only effectual method of withdrawing the contaminated air is by rarefaction*, a point not difficult of attainment in large establishments at all seasons of the year, and which can be accomplished in various ways. But, if a large and constant supply of pure air of a proper temperature be provided, and this can be readily effected by the air-pump invented by Dr. Arnott, less precautions will suffice for the removal of the carbonic acid and other noxious gases generated in hospitals, prisons, &c.; inasmuch as it is apparent, that fresh air can only be driven into an apartment by displacing an equal volume of the gaseous fluids contained in it.

It would not be doing justice to M. Lévy, if we did not recommend his treatise to the careful notice of our readers, feeling assured that it will be found, and especially at the present time, to be a valuable record of the important and diversified facts bearing on the preservation of health, and on the prevention of disease.

